# Clinical Differences between Eosinophilic and Noneosinophilic Acute Exacerbation of Chronic Obstructive Pulmonary Disease: A Multicenter Cross-Sectional Study

**DOI:** 10.1155/2020/1059079

**Published:** 2020-11-12

**Authors:** Guangming Dai, Yajuan Ran, Jiajia Wang, Xingru Chen, Junnan Peng, Xinglong Li, Huojin Deng, Min Xiao, Tao Zhu

**Affiliations:** ^1^Respiratory Department, First People's Hospital of Suining City, 629000 Suining, Sichuan, China; ^2^Pharmacy Department, Second Affiliated Hospital of Chongqing Medical University, 400010 Chongqing, China; ^3^Rheumatology Medicine, Second Affiliated Hospital of Chongqing Medical University, 400010 Chongqing, China; ^4^Respiratory Medicine, Second Affiliated Hospital of Chongqing Medical University, 400010 Chongqing, China; ^5^Respiratory Medicine, Zhujiang Hospital of Southern Medical University, Guangzhou 510280, China; ^6^Respiratory Medicine, and Division of Pulmonary Diseases, State Key Laboratory of Biotherapy of China, West China Hospital of Sichuan University, Chengdu 610041, China

## Abstract

**Methods:**

A total of 643 AECOPD patients were enrolled in this multicenter cross-sectional study. Finally, 455 were included, 214 in the normal-eosinophil AECOPD (NEOS-AECOPD) group, 63 in the mild increased-eosinophil AECOPD (MEOS-AECOPD) group, and 138 in the severe increased-eosinophil AECOPD (SEOS-AECOPD) group. Demographic data, underlying diseases, symptoms, and laboratory findings were collected. Multiple logistic regression analysis was performed to identify the independent factors associated with blood eosinophils (EOS). Correlations between blood EOS and its associated independent factors were evaluated.

**Results:**

The significant differences in 19 factors, including underlying diseases, clinical symptoms, and laboratory parameters, were identified by univariate analysis. Subsequently, multiple logistic regression analysis revealed that lymphocyte%, neutrophil% (NS%), procalcitonin (PCT), and anion gap (AG) were independently associated with blood EOS in AECOPD. Both blood EOS counts and EOS% were significantly correlated with lymphocyte%, NS%, PCT, and AG.

**Conclusions:**

Collectively, blood EOS was independently associated with lymphocyte%, NS%, PCT, and AG in AECOPD patients. Lymphocyte% was lower, and NS%, PCT, and AG were higher in eosinophilic AECOPD. Our results indicate that viral-dominant infections are the probable major etiologies of eosinophilic AECOPD. Noneosinophilic AECOPD is more likely associated with bacterial-dominant infections. The systemic inflammation in noneosinophilic AECOPD was more severe.

## 1. Introduction

Chronic obstructive pulmonary disease (COPD) is the most common chronic pulmonary disorder. It is found that the prevalence of COPD is gradually increasing in recent decades [1–3]. Wang *et al.* showed that the prevalence of COPD was 8.6%, indicating about 99.9 million patients in mainland China [3]. It is estimated that about 3.2 million people died from COPD worldwide in 2015 [2]. Globally, COPD is the third leading cause of death in recent years [1, 4]. Furthermore, COPD is a highly heterogeneous disease with different responses and outcomes [5, 6].

Although other potential biomarkers were identified [7] such as inflammatory mediators/proteins [8], miRNAs [9, 10], DNA methylation CpG sites [11, 12], single nucleotide polymorphisms [13, 14], and metabolites [15, 16], blood eosinophils (EOS) are considered to be stable, easily available, and acceptable markers in clinical practice [17, 18]. Generally, COPD is considered a Th1-dependent chronic airway inflammation. Neutrophils (NS), macrophages, and Th1 cells are the major immunological cells in COPD, whereas EOS, B cells, and Th2 cells are essential for asthma [1, 19–21]. However, evidence has proven that EOS are also increased in a group of COPD patients (not only in blood but also in sputum) and that higher blood EOS are associated with increased risk of readmission, severe lung function impairment, and longer hospital stay (LHS) [17, 22–26]. Some studies identified that inhaled corticosteroid (ICS) plus long-acting *β*2-agonist (LABA) and ICS plus LABA and long-acting muscarinic antagonist (LAMA) brought more benefits in eosinophilic COPD than in noneosinophilic COPD [27, 28]. Therefore, increased blood EOS was considered to be a “treatable trait” of COPD [18, 25]. Nevertheless, the clinical features of eosinophilic hospitalized AECOPD are still not well studied. Thus, this study was aimed at exploring the clinical differences between eosinophilic and noneosinophilic AECOPD.

**Figure 1:**
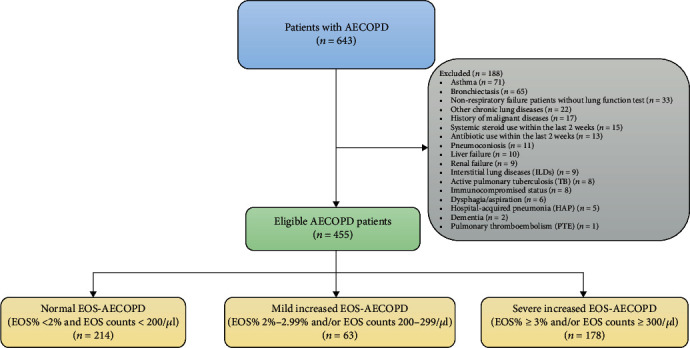
Flow diagram of the study.

Additionally, the optimal cutoff value of blood EOS is still not determined. With the cutoff of EOS% ≥ 2% and/or EOScounts ≥ 200 cells/*μ*L, Couillard *et al.* showed that the risk of 12-month COPD-related readmission in eosinophilic AECOPD was increased and LHS was not different, as compared to noneosinophilic AECOPD [22]. With a cutoff of 300 cells/*μ*L, Qi et al. found that sputum microbiome richness and plasma IL-6 levels in eosinophilic AECOPD decreased more significantly than in noneosinophilic AECOPD, after 7 days of treatment [29]. Cheng and Lin demonstrated that the ICS response in COPD with EOS% > 3% was better than that in noneosinophilic COPD [30]. Therefore, in our study, the patients with AECOPD were divided into three subgroups considering both blood EOS counts and EOS% (Figure 1).

## 2. Methods

### 2.1. Study Design and Population

This multicenter cross-sectional study was performed at the Respiratory Department of the Second Affiliated Hospital of Chongqing Medical University and the First People's Hospital of Suining City from January 2017 to January 2020. This study was approved by the Research Ethics Committees of the Second Affiliated Hospital of Chongqing Medical University (No. 2019-23) in accordance with the Declaration of Helsinki. Informed consent was obtained from all patients by the responsible physician or an appropriately trained staff member. Standard care and treatments were provided according to current clinical guidelines [1, 5].

### 2.2. Sample Size Determinations

As for the sample size, a minimum total of 159 (53 in each group) was required to detect at least a 25% difference in effect size for an 80% power, assuming *α* = 0.05 and allocationratio = 1 : 1 : 1. Furthermore, 20% more patients (64 in each group) were recruited.

### 2.3. Inclusion and Exclusion Criteria

The inclusion criterion was COPD exacerbation requiring hospitalization with age ≥ 40 years. Exclusion criteria were as follows (in descending order): asthma (*n* = 71), bronchiectasis (*n* = 65), nonrespiratory failure patients without lung function test (*n* = 33), other chronic lung diseases (*n* = 22), history of malignant diseases (*n* = 17), systemic steroid use within the last 2 weeks (*n* = 15), antibiotics use within the last 2 weeks (*n* = 13), pneumoconiosis (*n* = 11), liver failure (*n* = 10), renal failure (*n* = 9), interstitial lung diseases (ILDs) (*n* = 9), active pulmonary tuberculosis (TB) (*n* = 8), immunocompromised status (organ transplant, immunosuppressive agent use, and HIV infection) (*n* = 8), dysphagia and aspiration (*n* = 6), hospital-acquired pneumonia (HAP) (*n* = 5), dementia (*n* = 2), and pulmonary thromboembolism (PTE) (*n* = 1). A total of 643 patients with hospitalized AECOPD were recruited, of which 188 were excluded. In the end, 214 were NEOS-AECOPD patients, 63 were MEOS-AECOPD patients, and 178 were SEOS-AECOPD patients (Figure 1).

### 2.4. Definitions

According to the Global Initiative for Chronic Obstructive Lung Disease (GOLD) classification [1], the diagnosis of COPD was established by a pulmonologist based on a history of exposure to risk factors, such as smoking, biomass fuel exposure, and occupational dust; clinical presentations; and airflow obstruction measured by spirometry (a postbronchodilator fixed ratio of FEV1/FVC < 0.7). AECOPD was defined as an event in the natural course of the disease characterized by acute changes in clinical symptoms beyond normal day-to-day variation, resulting in additional therapy [1]. Connective tissue disease (CTD) was defined as having a previous rheumatologist diagnosis of a specific CTD, such as systemic lupus erythematosus, Sjogren's syndrome, systemic sclerosis, and rheumatoid arthritis. Both blood EOS counts and EOS% were considered to set the cutoff values of EOS. Normal-eosinophil AECOPD (NEOS-AECOPD) was defined as AECOPD with EOS% < 2% and EOScounts < 200 cells/*μ*L. Mild increased-eosinophil AECOPD (MEOS-AECOPD) was defined as AECOPD with EOS% 2%-2.99% and/or EOS counts 200-299cells/*μ*L. Severe increased-eosinophil AECOPD (SEOS-AECOPD) was defined as AECOPD with EOS% ≥ 3% and/or EOScounts ≥ 300 cells/*μ*L. Ex-smokers were defined as abstaining from smoking ≥ 6 months. Neutrophil-to-lymphocyte ratio (NLR) was defined as neutrophils divided by lymphocytes in the blood.

### 2.5. Data Collection

In our study, demographic data, underlying diseases, comorbid conditions, symptoms, and LHS were recorded and collected. Blood samples for laboratory tests and lung function tests were all collected and performed within 24 h after admission. However, for safety reasons and cooperation concerns, the spirometer test was not performed in patients with respiratory failure. All patients underwent high-resolution computed tomography (HRCT) scans within 48 h of hospitalization, and the results were reviewed by one independent radiologist and one pulmonologist in each hospital. Additionally, the participating centers shared the same methodologies and normal values in the laboratory measurements.

### 2.6. Statistical Analysis

Data were analyzed using SPSS 20.0 software (SPSS Inc., Chicago, IL, USA). Continuous variables were expressed as the mean ± standarddeviation (SD), and categorical data were expressed as frequencies. The data distribution was analyzed using the Kolmogorov–Smirnov test. Continuous variables with normal distribution were analyzed by one-way ANOVA with LSD and SNK's post hoc test. Continuous variables with abnormal distribution and ordinal variables were measured using the Kruskal–Wallis *H* test. The chi-squared test was used to analyze categorical variables. Multiple logistic regression was performed to investigate the independent risk factors associated with blood EOS in AECOPD patients. The Spearman rank correlation coefficient was calculated to analyze correlations. A threshold of *P* < 0.05 was considered to be significant.

## 3. Results

### 3.1. Baseline Characteristics of AECOPD Patients

A total of 643 hospitalized patients with AECOPD were screened (Figure 1). Finally, 214 (47.03%) NEOS-AECOPD patients, 63 (13.85%) MEOS-AECOPD patients, and 178 (39.12%) SEOS-AECOPD patients were eligible. The ratio of eosinophilic AECOPD (MEOS-AECOPD+SEOS-AECOPD) was 52.97%. The demographic data of the patients are shown in Table 1. The rate of CTD was significantly higher in SEOS-AECOPD patients.

### 3.2. Clinical Features and Laboratory Data of AECOPD Patients

As shown in Table 2, the rates of fever and mechanical ventilation (MV), white blood cells (WBCs), neutrophils (NS), NS%, lymphocyte%, NLR, procalcitonin (PCT), C-reactive protein (CRP), erythrocyte sedimentation rate (ESR), anion gap (AG), serum sodium (Na^+^), serum potassium (K^+^), serum calcium(Ca^2+^), serum magnesium (Mg^2+^), blood urea nitrogen (BUN), direct bilirubin (DBIL), and LHS were significantly different among the three groups.

### 3.3. Multiple Logistic Regression Analysis in AECOPD Patients

To explore independent factors associated with blood EOS in AECOPD patients, multiple logistic regression analysis was performed. In the multiple logistic regression model, the factors significantly associated with blood EOS in univariate analysis, including the rates of CTD, fever, MV, WBC, NS, NS%, lymphocytes%, NLR, PCT, CRP, ESR, AG, serum Na^+^, serum K^+^, serum Ca^2+^, serum Mg^2+^, BUN, DBIL, and LHS, were included. As shown in Table 3, lymphocyte%, NS%, PCT, and AG were independently associated with blood EOS in AECOPD patients by multiple logistic regression.

### 3.4. Correlations between Blood EOS Counts/EOS% and Lymphocyte%, NS%, PCT, and AG in AECOPD Patients

Since lymphocyte%, NS%, PCT, and AG were independently associated with blood EOS in AECOPD patients, their correlations with blood EOS counts and EOS% were explored. Significant correlations were found between blood EOS counts and lymphocyte%, NS%, PCT, and AG and between blood EOS% and lymphocyte%, NS%, PCT, and AG in AECOPD patients (Table 4). Among them, lymphocyte% was positively and NS%, PCT, and AG were negatively correlated with blood EOS counts and EOS%.

## 4. Discussion

In this multicenter cross-sectional study, we found that lymphocyte%, NS%, PCT, and AG were the independent factors associated with blood EOS in AECOPD patients. Our results indicate that viral-dominant infection is probably related to eosinophilic AECOPD. Noneosinophilic AECOPD is more likely to be associated with bacterial-dominant infections.

As the most common lung disorder, the prevalence of COPD is still increasing [1, 3, 5]. Globally, the prevalence of COPD was 11.7% (8.4%-15.0%), and the COPD case number was approximately 384 million in 2010 [1, 4]. Wang *et al.* showed that the overall prevalence of COPD in mainland China was 8.6% (95% CI 7.5-9.9) in a population aged ≥40 years or approximately 99.9 (95% CI, 76.3-135.7) million cases [3]. Simultaneously, COPD is a chronic disease with high mortality and disability. It was reported that approximately 3 million people die from COPD every year [1, 31]. Patel *et al.* showed that COPD caused an average of 5 more days of work absence and short-term disability-associated extra costs of $641 each year in the USA [32]. It was estimated that the number of years living with disability of COPD was about 29.4 million in 2010 [33].

Nevertheless, COPD is a highly heterogeneous disease with significant differences in treatment response and outcomes in patients. Mounting evidence suggests that individual therapy and target therapy are the major trends of COPD in the future. Therefore, exploration and differentiation of the phenotypes of COPD are valuable in clinical practice. Recently, a number of studies have shown that blood EOS (EOS counts and EOS%) are an effective, stable, and available biomarkers in COPD and can be used to define the phenotypes of COPD [17, 22, 34, 35]. However, the cutoff value of blood EOS is still debated, ranging from 150 to 400 cells/*μ*L and/or 2% to 4% in different studies [17, 18, 22, 23, 25, 34–36]. Therefore, in this study, both 200 cells/*μ*L and 300 cells/*μ*L and 2% and 3% were considered the cutoff values of blood EOS counts and EOS% in AECOPD patients (Figure 1). According to demographic data, no differences in sex, age, BMI, smoking status, lung functions (GOLD stages), and most comorbidities and complications were found among the three groups (Table 1). Only the rate of CTD was significantly different among the three groups. The rate of CTD in the SEOS-AECOPD group was higher than that in the NEOS- and MEOS-AECOPD groups (Table 1). These results indicate that sex, BMI, smoking, and lung function were not associated with blood EOS in AECOPD patients.

The correlations between blood EOS and lung EOS (induced sputum, bronchoalveolar lavage fluid (BALF), and tissues) were still controversial. Many studies showed that blood EOS was considered to be a good predictor of EOS in airways in COPD patients [23, 37, 38]. Eltboli *et al.* identified a strong correlation between blood EOS% and the submucosal EOS count (*r* = 0.57) and reticular basement membrane thickness (*r* = 0.59) in COPD patients [37]. Kolsum *et al.* reported that compared with COPD with blood EOS < 150 cells/*μ*L, EOS% in induced sputum, BALF, and submucosa were all higher in COPD with blood EOS > 300 cells/*μ*L [38]. Nevertheless, several studies found that the correlation between lung EOS and blood EOS was not very well [36, 39]. Turato *et al.* explored the correlations between blood EOS and EOS in central airways, peripheral airways, and lung parenchyma, using samples of COPD patients who underwent lung resection for solitary pulmonary nodules without additional complications [36]. Initially, no differences in EOS densities among central airways, peripheral airways, and lung parenchyma were observed in COPD, and pulmonary EOS counts were not associated with COPD severity. Subsequently, they revealed that the correlations between blood EOS and EOS in any of the three lung compartments were not significant. Additionally, in a randomized, double-blind, placebo-controlled trial, EOS counts and EOS% in induced sputum were markedly reduced after 16 weeks of roflumilast (a PDE4 inhibitor) treatment in COPD [39]. However, blood EOS counts were not changed by roflumilast. Meanwhile, a significant difference was confirmed between eosinophilic and noneosinophilic AECOPD [17, 20, 22, 24, 25, 30, 35, 40]. Mounting evidence has shown that increased blood EOS was associated with higher risk of readmission, severe lung function impairment, longer LHS and survival time, and better ICS response in COPD patients [22, 24, 25]. Nevertheless, the clinical features, particularly laboratory parameters, of eosinophilic AECOPD are still not well studied. In this study, commonly used laboratory parameters, including blood routine, PCT, ESR, CRP, ABG, electrolytes, liver function, and renal function, were included. Our data showed that the rates of fever and MV, WBC, NS, NS%, lymphocyte%, NLR, PCT, CRP, ESR, AG, serum Na^+^, serum K^+^, serum Ca^2+^, serum Mg^2+^, BUN, DBIL, and LHS were significantly different among the three groups (Table 2). Subsequently, 19 variables with significant differences in univariate analysis were included in the multiple logistic regression analysis. We identified that lymphocyte%, NS%, PCT, and AG were independently associated with blood EOS in AECOPD patients.

Furthermore, as shown in Table 4, lymphocyte% was positively and NS%, PCT, and AG were negatively correlated with blood EOS counts and EOS% in AECOPD. In this study, asthma was strictly excluded, which was considered to be the most common confounder of COPD studies [18, 24, 40]. Meanwhile, COPD patients with recent systemic steroid and immunosuppressive agent use were also excluded. These data indicate that inflammatory types are significantly different between eosinophilic and noneosinophilic AECOPD patients. EOS and lymphocytes were the major inflammatory cells in eosinophilic AECOPD, and neutrophils were the dominant inflammatory cells in noneosinophilic AECOPD. It is well known that respiratory tract infection is the leading cause of acute exacerbation in COPD [1, 35, 41–43]. Among them, bacteria and viruses are the most common pathogens. In a prospective observational study, Bafadhel *et al.* showed that 55% and 29% of acute exacerbations were related to bacterial and viral infections in COPD [43]. Meanwhile, Papi *et al.* demonstrated that bacterial and/or viral infection was found in 78.1% (29.7% bacterial, 23.4% viral, and 25% viral/bacterial coinfection) AECOPD patients [41]. Several studies have shown that airway eosinophilic inflammation is related to viral infection in AECOPD [35, 41]. Additionally, it was confirmed that blood neutrophils and PCT are biomarkers of bacterial infection in COPD [44]. In a meta-analysis, Ni *et al.* showed that the sensitivity and specificity of PCT in diagnosing bacterial infections were 0.60 and 0.76, respectively, and the AUC of the ROC curve was 0.77 [44]. Ergan *et al.* found that compared with culture-negative patients, PCT was markedly increased in culture-positive patients in AECOPD [45]. They also showed that 0.25 ng/mL was the optimal cutoff value, with 63% sensitivity, 67% specificity, and 0.73 AUC, to predict bacterial infection in AECOPD. Collectively, our results suggest that viral and virus-dominant infections are probably the major etiologies of eosinophilic acute exacerbation in COPD. Noneosinophilic acute exacerbation in COPD is more likely associated with bacterial and bacterial-dominant infection.

Additionally, NS%, PCT, and AG were negatively correlated with blood EOS in AECOPD patients (Table 4). Moreover, AG, PCT, and NS% in SEOS-AECOPD were significantly lower than those in NEOS-AECOPD and MESO-AECOPD (Table 2). No difference in metabolic acidosis was observed among the three groups (Table 1). Circulation and tissue hypoperfusion are associated with severe infection in clinical practice. Commonly, hypoperfusion-induced hyperlactacidemia is the major reason for increased AG in patients with infection without renal failure and ketoacidosis. Durmuş *et al.* revealed that lactate clearance in hospitalized AEOPD patients (severe patients) was significantly lower than that in AECOPD patients without hospitalization (nonsevere patients) [46]. These results collectively indicate that bacterial infection and systemic inflammation in noneosinophilic AECOPD are more severe than in eosinophilic AECOPD.

Due to low positive rates of sputum cultures, specimen contamination, and airway bacterial colonization in COPD patients, the pathogen results were not included to reduce biases and confounders, which was also one of the major limitations of our current study. Therefore, direct correlations between pathogen types and blood EOS were not evaluated. The main strength of our study was that relatively comprehensive laboratory data were collected, which accurately classified the severity and complications of the underlying diseases. In particular, a chest HRCT scan was performed in each patient, which effectively promoted the diagnosis accuracy and excluded most other lung diseases. Furthermore, the different cutoff values of blood EOS were considered, making our data more convincing.

## 5. Conclusions

Collectively, our results revealed that lymphocyte%, NS%, PCT, and AG were independent factors associated with blood EOS in AECOPD patients. Our data indicated that viral and viral-dominant infections are probably the major etiologies of eosinophilic AECOPD. Noneosinophilic AECOPD is more likely to be associated with bacterial and bacterial-dominant infections. Systemic inflammation in noneosinophilic AECOPD is more severe than in eosinophilic AECOPD. Nevertheless, further studies with high sensitivity and specificity in pathogen tests, including bronchoscopy, should be developed to validate our results.

## Figures and Tables

**Table 1:** Demographic data of the patients with AECOPD (*n* = 455).

	NEOS-AECOPD (*n* = 214)	MEOS-AECOPD (*n* = 63)	SEOS-AECOPD (*n* = 178)	Statistical values	*P*
Sex (male, *n*)	159	51	138	1.375	0.503
Age (years)	71.2056 ± 9.31175	73.1429 ± 9.89437	70.2528 ± 9.06961	2.272	0.104
BMI	21.908271 ± 3.6468114	22.739524 ± 3.3535515	22.285506 ± 4.0060567	1.329	0.266
Smoking				0.366	0.833
Nonsmoking	74	24	71		
Ex-smoking	56	11	36		
Current smoking	84	28	71		
GOLD stages				5.875	0.053
Stage I: mild (≥80%)	25	10	21		
Stage II: moderate (50-79%)	62	24	59		
Stage III: severe (30-49%)	54	17	52		
Stage IV: very severe (<30%) without respiratory failure	17	4	13		
Respiratory failure	56	8	33		
Underlying diseases/comorbidities					
Pneumothorax	2	0	3	1.317	0.518
Pleural effusion (PE)	11	0	6	3.684	0.158
Community-acquired pneumonia (CAP)	96	26	74	0.525	0.769
Cor pulmonale	43	6	26	4.698	0.095
Coronary artery disease (CAD)	46	12	27	2.567	0.277
Hypertension	80	21	73	1.288	0.525
T2DM	39	14	20	5.595	0.061
Atrial fibrillation (Af)	11	3	5	1.383	0.501
Connective tissue disease (CTD)	0	0	4	6.280	0.043
Metabolic acidosis	35	12	20	3.116	0.211

**Table 2:** Clinical features and laboratory data of the patients with AECOPD (*n* = 455).

	NEOS-AECOPD (*n* = 214)	MEOS-AECOPD (*n* = 63)	SEOS-AECOPD (*n* = 178)	Statistical values	*P*
Fever	29	0	7	18.622	0.000
WBCs (×10^9^/L)	9.127710 ± 4.0336889	6.900000 ± 3.3279302	7.207528 ± 2.5874116	19.396	0.000
NS (×10^9^/L)	7.495327 ± 6.4450553	4.818889 ± 3.1813231	4.869382 ± 2.1464153	17.341	0.000
Lymphocytes (×10^9^/L)	1.365327 ± 0.9051292	1.470476 ± 0.6305692	1.479326 ± 0.5649365	1.253	0.287
EOS (×10^9^/L)	0.059393 ± 0.0508252	0.156032 ± 0.0493683	0.502022 ± 0.5874828	71.795	0.000
NS%	78.291729 ± 37.4499608	67.818413 ± 9.5222841	66.115618 ± 10.2687442	11.008	0.000
Lymphocyte%	16.887710 ± 9.9761204	23.018254 ± 7.9939971	21.994551 ± 8.6718274	19.520	0.000
EOS%	0.747570 ± 0.6408311	2.359206 ± 0.3470767	6.685562 ± 5.0527810	170.959	0.000
NLR	7.945654 ± 9.5498819	4.191587 ± 5.7973724	3.699944 ± 2.0624385	19.620	0.000
RBCs (×10^12^/L)	4.416748 ± 0.7819829	4.535873 ± 0.5766411	4.525337 ± 0.6074892	1.474	0.230
Hb (g/L)	132.35514 ± 17.8379520	135.31746 ± 14.2464771	134.23118 ± 18.3276071	0.944	0.390
Hct (%)	40.175467 ± 5.3567446	40.890476 ± 3.9642643	40.921348 ± 4.3530357	1.337	0.264
PLTs (×10^9^/L)	203.242991 ± 76.3426106	186.555556 ± 60.5199988	201.404494 ± 73.6012649	1.301	0.273
PCT (ng/mL)	0.218766 ± 0.7493592	0.139349 ± 0.6293068	0.065798 ± 0.0805874	3.545	0.030
CRP (mg/mL)	27.364953 ± 40.8671745	16.965079 ± 28.8966242	17.563483 ± 29.1130111	4.563	0.011
ESR (mm/first hour)	24.635514 ± 20.9070793	19.714286 ± 20.5578872	19.932584 ± 19.6588404	3.095	0.046
ABG					
pH	7.412682 ± 0.2815835	7.431905 ± 0.0361385	7.429663 ± 0.0477150	0.458	0.633
PaCO_2_ (mmHg)	43.564486 ± 13.6961751	40.746032 ± 6.5302479	42.502247 ± 9.1182623	1.600	0.203
PaO_2_ (mmHg)	82.598131 ± 28.5118738	79.777778 ± 19.4492262	78.938202 ± 21.5099925	1.115	0.329
Oxygen index (OI)	343.990654 ± 97.8380856	347.984127 ± 72.4307215	346.898876 ± 85.3106050	0.075	0.928
AB (mmol/L)	28.489252 ± 6.1881931	26.898413 ± 3.8558080	27.778090 ± 4.0188008	2.582	0.077
SB (mmol/L)	27.662150 ± 3.4513272	26.823810 ± 2.7911562	27.159551 ± 2.3971602	2.505	0.083
AG	12.104299 ± 4.3290494	12.295238 ± 4.0810905	9.645506 ± 5.4451873	14.966	0.000
Serum Na^+^ (mmol/L)	137.636916 ± 5.0665516	139.073016 ± 4.0107198	138.876966 ± 4.4745202	4.308	0.014
Serum K^+^ (mmol/L)	3.896262 ± 0.4741605	3.965556 ± 0.3839051	4.016742 ± 0.0303221	3.737	0.025
Serum Ca^2+^ (mmol/L)	2.223738 ± .1488486	2.242222 ± 0.1972917	2.268483 ± 0.1781526	3.452	0.033
Serum Mg^2+^ (mmol/L)	0.839720 ± 0.0951594	0.886032 ± 0.1219669	0.870730 ± 0.1146699	6.539	0.002
ALB (g/L)	37.813551 ± 4.3179452	37.894127 ± 3.9404780	38.765169 ± 4.1056968	2.703	0.068
BUN (mmol/L)	6.562897 ± 2.5492516	6.708571 ± 2.3177268	5.946236 ± 1.8600346	4.539	0.011
Cr (*μ*mol/L)	72.299533 ± 27.8074238	73.471587 ± 23.2868408	73.812865 ± 20.2982227	0.196	0.822
ALT (U/L)	24.677570 ± 38.0325258	26.728571 ± 43.6346209	21.196629 ± 22.0215762	0.836	0.434
AST (U/L)	27.925234 ± 43.1549672	28.396825 ± 45.6301894	23.117978 ± 16.9972617	1.030	0.358
IBIL (*μ*mol/L)	6.397243 ± 3.3865077	6.846032 ± 3.6854914	5.984831 ± 2.8904372	1.826	0.162
DBIL (*μ*mol/L)	5.060234 ± 3.0660336	4.557143 ± 2.8291521	4.007865 ± 1.7511723	8.000	0.000
RBG (mmol/L)	6.878692 ± 2.8710151	6.809048 ± 2.3441855	6.508539 ± 1.9257216	1.136	0.322
LHS (days)	9.9112 ± 4.90727	9.2381 ± 4.02698	8.6180 ± 3.80605	4.233	0.015
MV				8.671	0.013
Nonventilation	187	59	170		
NIPPV	26	4	8		
IPPV	1	0	0		

Abbreviations: WBCs: white blood cells; NS: neutrophils; EOS: eosinophils; NLR: neutrophil-to-lymphocyte ratio; RBCs: red blood cells; PLTs: platelets; Hb: hemoglobin; Hct: hematocrit; PCT: procalcitonin; CRP: C-reactive protein; ESR: erythrocyte sedimentation rate; ABG: air blood gas; AB: actual base; SB: standard base; AG: anion gap; Na^+^: sodium; K^+^: potassium; Ca^2+^: calcium; Mg^2+^: magnesium; ALB: albumin; Cr: creatinine; BUN: blood urea nitrogen; ALT: alanine aminotransferase; AST: aspartate aminotransferase; IBIL: indirect bilirubin; DBIL: direct bilirubin; RBG: random blood glucose; LHS: length of hospital stay; MV: mechanical ventilation; NIPPV: noninvasive positive pressure ventilation; IPPV: invasive positive pressure ventilation.

**Table 3:** Multiple logistic regression analysis of independent factors associated with blood eosinophils in AECOPD (*n* = 455).

	Estimate	S.E	Wals	df	Sig.	95% CI
Lymphocyte%	-0.238	0.037	41.127	1	0.000	-0.311~-0.165
NS%	-0.254	0.041	38.431	1	0.000	-0.334~-0.174
PCT	-1.494	0.355	17.739	1	0.000	-2.189~-0.799
AG	-0.099	0.022	19.587	1	0.000	-0.142~-0.055

Abbreviations: NS: neutrophils; PCT: procalcitonin; AG: anion gap.

**Table 4:** The correlations between EOS counts/EOS% in blood and lymphocyte%, NS%, PCT, and AG in AECOPD patients (*n* = 455).

	Lymphocyte%	NS%	PCT	AG
EOS counts				
*R*	0.221	-0.365	-0.214	-0.184
*P*	0.000	0.000	0.000	0.000
EOS%				
*R*	0.335	-0.481	-0.262	-0.222
*P*	0.000	0.000	0.000	0.000

Abbreviations: NS: neutrophils; PCT: procalcitonin; AG: anion gap.

## Data Availability

Due to the respect to and the protection of patient privacy, the data generated and/or analyzed in this study are not publicly available. However, they are available from the corresponding authors on reasonable request.
